# Comparison of Outcomes among Adult Patients with Nosocomial Bacteremia Caused by Methicillin-Susceptible and Methicillin-Resistant *Staphylococcus aureus*: A Retrospective Cohort Study

**DOI:** 10.1371/journal.pone.0144710

**Published:** 2015-12-21

**Authors:** Jann-Tay Wang, Le-Yin Hsu, Tsai-Ling Lauderdale, Wen-Chien Fan, Fu-Der Wang

**Affiliations:** 1 Department of Internal Medicine, National Taiwan University Hospital, Taipei, Taiwan; 2 Institute of Epidemiology and Preventive Medicine, National Taiwan University, Taipei, Taiwan; 3 National Institute of Infectious Diseases and Vaccinology, National Health Research Institutes, Zhunan, Taiwan; 4 Department of Internal Medicine, Taipei Veterans General Hospital, Taipei, Taiwan; 5 National Yang-Ming University School of Medicine, Taipei, Taiwan; University Hospital Münster, GERMANY

## Abstract

Several studies have shown that patients with bacteremia caused by methicillin-resistant *Staphylococcus aureus* (MRSA) have worse outcomes than those with bacteremia caused by methicillin-susceptible *S*. *aureus* (MSSA). However, only a limited number of studies have stratified the MRSA isolates into healthcare-associated (HA-) and community-associated (CA-) MRSA strains in such a comparison. This three-year retrospective cohort study, enrolling adult patients with nosocomial *S*. *aureus* bacteremia (SAB), was designed to investigate whether CA-MRSA and/or HA-MRSA strains were associated with different outcomes in comparison to MSSA in such a setting. The drug susceptibilities and staphylococcal cassette chromosome *mec* (SCC*mec*) types were determined for all of the causative isolates available. The MRSA bacteremia was further categorized into those caused by CA-MRSA strains (CA-MRSA-S bacteremia) when the causative isolates carried the type IV or V SCC*mec* element, those caused by HA-MRSA strains (HA-MRSA-S bacteremia) when the isolates carried the type I, II, or III SCC*mec* element, or unclassified MRSA bacteremia when the isolates were not available. The relevant demographic, clinical, and laboratory data were collected by reviewing the patients’ charts. The primary outcome was all-cause in-hospital mortality. A total of 353 patients were studied. The overall in-hospital mortality rate was 32.6%, with 23.3% in MSSA, 30.5% in CA-MRSA-S, 47.5% in HA-MRSA-S, and 35.3% in unclassified MRSA bacteremia, respectively. The multivariate analysis showed that HA-MRSA-S, but not CA-MRSA-S, bacteremia was associated with a significantly worse outcome compared with MSSA. The other risk factors independently associated with all-cause in-hospital mortality included the Charlson co-morbidity index, septic shock, thrombocytopenia, and persistent bacteremia. Resistance to linezolid and daptomycin was found among the MRSA isolates. The present study showed that bacteremia caused by HA-MRSA-S, but not CA-MRSA-S, was an independent risk factor for all-cause in-hospital mortality in patients with nosocomial SAB. Continuous monitoring regarding the susceptibilities of MRSA to linezolid and daptomycin is necessary.

## Introduction

*Staphylococcus aureus* is an important human pathogen that causes several serious infection syndromes in both community- and healthcare-associated settings [1]. Among these infection syndromes, *S*. *aureus* bacteremia (SAB) is of greatest concern because it is associated with significant mortality and morbidity [2, 3]. When treating *S*. *aureus* infections, resistance to methicillin poses an additional challenge, because methicillin-resistant *S*. *aureus* (MRSA) infections do not respond to most β-lactams, which are the most commonly used empirical antibiotics [4]. This, in turn, leads to delays in initiating effective antibiotic treatment.

MRSA was identified in 1961 and became wide-spread at the beginning of the 1980s [5, 6]. Traditionally, before the 1990s, nearly all MRSA infections were healthcare-associated and developed particularly in patients with various underlying medical conditions [7]. Since the 1990s, a new type of MRSA that could cause infections among previously healthy people in the community setting was noted, and was named community-associated MRSA (CA-MRSA) to differentiate it from the traditional healthcare-associated MRSA (HA-MRSA) [6, 7]. In the 1990s, the new type CA-MRSA infections were typically caused by MRSA strains carrying the type IV or V staphylococcal cassette chromosome *mec* (SCC*mec*) element (CA-MRSA strains), whereas the traditional HA-MRSA infections were caused by MRSA strains carrying type I, II, or III SCC*mec* element (HA-MRSA strains) [7, 8]. CA-MRSA strains (CA-MRSA-S) have later invaded into healthcare facilities and they have even replaced HA-MRSA strains (HA-MRSA-S) to cause a significant portion of healthcare-associated infections (HAIs) [9, 10]. However, patient factors in those with HAIs caused by CA-MRSA-S still differed from those with HAIs caused by HA-MRSA-S [11, 12]. In these comparisons, patients with HAIs caused by CA-MRSA-S tended to have earlier disease onsets following admission, to be younger, and to have fewer underlying diseases.

Several studies have demonstrated that MRSA bacteremia is associated with a significantly higher mortality rate compared with methicillin-susceptible *S*. *aureus* (MSSA) bacteremia [13, 14]. The higher level of mortality is thought to be related to delays in administering effective antimicrobial treatment, differences in the intrinsic virulence of the microbes, the slower bactericidal effect of glycopeptides compared to β-lactams against *S*. *aureus* infections, and host factors [4, 14]. However, only a limited number of studies stratified the causative MRSA isolates into CA-MRSA-S and HA-MRSA-S [6–8, 15, 16]. Whether the differences in the mortality rates between MSSA and MRSA bacteremia are general or specific to patients with MSSA bacteremia and those with bacteremia by CA-MRSA-S or between patients with MSSA bacteremia and those with bacteremia by HA-MRSA-S remain unclear.

Taiwan is an area where MRSA is highly prevalent in healthcare-associated settings, reaching as high as over 80% at one point [17]. Given this epidemiological background, patients with HAIs caused by gram-positive bacteria are usually administered glycopeptides empirically. Hence, the delay in initiating effective antibiotic treatment against MRSA, which has been considered a possible factor that contributes to the higher mortality rate in patients with MRSA bacteremia, is negligible among patients with SAB in healthcare-associated settings.

This study retrospectively collected the clinical data from patients with nosocomial SAB at two major teaching hospitals in Taiwan. Our primary aim was to compare all-cause in-hospital mortality among patients with nosocomial bacteremia caused by MSSA, CA-MRSA-S, and HA-MRSA-S.

## Materials and Methods

### Patients and data collection

From January 1, 2011 to December 31, 2013, all adult patients who were aged > 18 years and had been admitted to Taipei Veterans General Hospital (TVGH), which is a major tertiary teaching hospital with 2900 beds located in northern Taiwan, and National Taiwan University Hospital (NTUH), which is another major tertiary teaching hospital with 2500 beds located in northern Taiwan, with nosocomial SAB and no concomitant infections were retrospectively enrolled to participate in this study. Nosocomial *S*. *aureus* bacteremia was defined as ≥1 culture from a blood sample obtained 48 hours after admission and collected at the time of a fever of ≥ 38°C) yielding *S*. *aureus* [18]. If a patient had two separate episodes of SAB, (positive blood cultures for *S*. *aureus* 30 or more days after the prior positive blood culture [4]) during the study period, only the first episode was considered in the present study. Concomitant infection was defined as presence of other bacteria isolated form the same blood sample simultaneously or blood samples collected during the treatment course for SAB, or active infection by other bacteria at other body sites requiring antibiotic treatment when SAB developed or during the treatment course for SAB. The blood *S*. *aureus* isolates that had been preserved by the Departments of Laboratory Medicine at TVGH and NTUH were obtained for subsequent microbiological studies, which were undertaken at the central laboratory at NTUH.

A standardized case report form was used to collect the patients’ demographic, clinical, and routine laboratory data, which included the patient’s age and sex, the primary foci of the SAB, the severity of the infections (presence or absence of shock within 24 hours of the onset of SAB) [19], the presence of underlying diseases, the Charlson co-morbidity indices [20], the serum levels of albumin, C-reactive protein (CRP), creatinine, alanine aminotransferase (ALT), hemoglobin, white blood cell (WBC) and platelet counts at the time of onset of SAB.

The study was carried out in accordance with the principles stated in the Declaration of Helsinki, and approved by the Institutional Review Boards at Taipei Veterans General Hospital (TVGH-2015-02-012BC) and National Taiwan University Hospital (NTUH-201011008RC). Both Review Boards approved to waive inform consent due to the retrospective study design and the research posing no more than minimal risk.

### Microbiological studies

All of the preserved and available bacterial isolates were re-identified and confirmed as *S*. *aureus* by Gram stain, catalase-activity tests, and coagulase latex agglutination tests. MRSA was identified using CHROMagar^TM^ MRSA plates. In vitro susceptibilities to erythromycin, clindamycin, gentamicin, oxacillin, tetracycline, trimethoprim/sulfamethoxazole (SXT), rifampin, ciprofloxacin, vancomycin, linezolid, and daptomycin were determined by minimum inhibitory concentrations (MICs) using the broth micro-dilution method [21]. The susceptibility test results were interpreted using the criteria provided by the Clinical Laboratory Standards Institute [22]. The SCC*mec* element types were determined and multilocus-sequence typing was performed as described previously [8, 10].

### Definitions

Concerning the infection foci, SAB was classified as that without identifiable foci (SABNF), intra-vascular catheter-associated SAB (IVC-SAB), and SAB with identifiable foci other than intra-vascular catheters (SABWF). The infection foci were determined according to the medical records, which should indicate that *S*. *aureus* was also cultured from the infection foci, and the criteria provided by Centers for Disease Control and Prevention, USA [18]. Significant hypoalbuminemia was defined as a serum albumin level of < 2.5 g/L. Impaired renal function was defined as a serum creatinine level of > 1.4 mg/dL. Clinically significant abnormal liver function was defined as a serum ALT level of > 200 U/L, that is, 5 times the normal upper limit. Anemia was defined as a hemoglobin level of < 11 g/dL. An abnormal WBC count was defined as > 12,000/μL or < 4,000/μL. Thrombocytopenia was defined as a platelet count of < 150,000/μL. An effective antibiotic was defined in accordance with the in vitro susceptibility test results. Recent operations were defined as operations that had been performed under local anesthesia within seven days or operations that had been performed under general anesthesia within 30 days before the onset of SAB [23]. Persistent bacteremia was defined as a positive blood culture after 7 days of effective antibiotic treatment [24]. CA-MRSA-S were defined as MRSA isolates that carried the type IV or V SCC*mec* elements, and HA-MRSA-S were defined as MRSA isolates that carried the type I, II, or III SCC*mec* elements [8, 25]. When the causative *S*. *aureus* isolates were available, the SAB was classified as MSSA, CA-MRSA-S, or HA-MRSA-S bacteremia based on the results of the microbiological studies and the definitions provided above. When the causative *S*. *aureus* isolates were not available but could be identified as MSSA from the medical records, the SAB was categorized as MSSA bacteremia. When the causative *S*. *aureus* isolates were not available but could be identified as MRSA from the medical records, the SAB was categorized as unclassified MRSA bacteremia, because the SCC*mec* element typing could not be performed.

### Statistical analyses

The study’s primary endpoint was all-cause in-hospital mortality. The continuous variables are either expressed as the means ± standard deviations (SD) and compared using Student’s *t* test, or they are expressed as the medians and the ranges and compared with the Wilcoxon rank sum test if their distributions were not normal. The categorical variables were compared using the chi-square test or Fisher’s exact test if their expected values were below 10. The risk factors for all-cause in-hospital mortality were identified using logistic regression models. All of the parameters were initially tested using univariate analysis, and those with *p* values of <0.20 were considered in the multivariate analysis. A linear regression model was used to identify parameters that had collinearity, and these were not considered simultaneously in the final multivariate analysis. Also, variables with missing value of > 30% would not be considered in the final multivariate analysis. A stepwise model comparison and Akaike’s information criterion were used to determine the best model for the analysis of the multiple variables. The multivariate analysis was conducted using three different models. The Model 1 stratified SAB into MSSA, CA-MRSA-S, HA-MRSA-S, and unclassified MRSA bacteremia. The Model 2 stratified SAB into MSSA (no SCC*mec* element) bacteremia, and bacteremia caused by MRSA with carriage of type II SCC*mec* element, those with type III SCC*mec* element, those with type IV SCC*mec* element, those with type V SCC*mec* element, and those with unknown SCC*mec* type. The other variables tested in Model 1 and 2 were the same. The Model 3 was similar to the Model 1, but variables of SAB severity, such as septic shock, thrombocytopenia, abnormal WBC count, CRP level, and persistent bacteremia, were excluded from the multivariate analysis. All of the statistical analyses were performed using SAS 9.2 (SAS Institute, Cary, NC, USA). All of the tests were two-tailed and a *p* value of <0.05 was considered statistically significant.

## Results

During the study period, a total 612 episodes of nosocomial SAB developed among 597 adult patients were identified (388 at NTUH and 224 at TVGH). There were 15 episodes (all at NTUH) considered as recurrent ones and another 244 episodes (108 at NTUH and 136 episodes at TVGH) with various concomitant infections, including 104 episodes of polymicrobial bacteremia and 67 concomitant with active pneumonia, 44 with active urinary tract infection, and 29 with active skin and soft tissue infection caused by other bacteria, excluded from the study. Among the remaining 353 episodes (developed in 353 patients) enrolled in the present study, the causative pathogens were available for 261. No misclassifications of methicillin resistance were noted between the medical records and the microbiological investigations that were performed in the present study for all 261 *S*. *aureus* isolates, comprising 101 MSSA, 59 CA-MRSA-S, and 101 belonging to HA-MRSA-S isolates. Fifty-eight additional episodes of MSSA bacteremia were identified from the medical records. There were additional 34 episodes of unclassified MRSA bacteremia.

The demographic, clinical, and microbiological data from the patients who were enrolled in the study are listed in Table 1. The mean ± SD age of the patients was 60.0±24.4 years. The male to female ratio was 0.60. The Charlson co-morbidity index was 4.7±3.2. Seventy-three (20.7%) patients had persistent *S*. *aureus* bacteremia. One hundred and fifty patients had SABNF, 156 had IVC-SAB, and 47 had SABWF. The infection foci of the 47 patients with SABWF included respiratory tract (16 patients), surgical wound (16), skin (12), and urinary tract (3). Fifty patients presented with septic shock. Nine patients had endocarditis that was diagnosed using echocardiography. Two hundred and seventy-eight patients received effective antibiotic treatment within 48 hours after the onset of SAB, including 141 MRSA bacteremic patients, of whom 81 used vancomycin and 60 used teicoplanin, and 137 MSSA bacteremic patients. The overall in-hospital mortality rate was 23.3% for MSSA, 30.5% for CA-MRSA-S, 47.5% for HA-MRSA-S, 35.3% for unclassified MRSA, 40.2% for all MRSA, and 32.6% for all *S*. *aureus* bacteremia, respectively.

**Table 1:** The demographic, clinical, and relevant microbiological data of the 353 patients with nosocomial *S*. *aureus* bacteremia by strains.

Variables	MSSA (N = 159)	CA-MRSA-S (N = 59)	HA-MRSA-S (N = 101)	Unclassified MRSA (N = 34)	Total (N = 353)	*p* ^a^
Age (years, mean±SD)	58.8±24.2	53.5±27.2	64.8±22.5	62.3±24.1	60.0±24.4	0.033
Gender (male-to-female)	59:100	22:37	34:67	17:17	132:221	0.405
Underlying diseases (%)						
Cardiovascular diseases	95 (60)	35 (59)	67 (66)	22 (65)	219 (62)	0.698
Respiratory diseases	36 (23)	24 (41)	39 (39)	11 (32)	110 (31)	0.015
Neurologic diseases	47 (30)	18 (31)	33 (33)	12 (35)	110 (31)	0.901
Gastrointestinal diseases	36 (23)	21 (36)	34 (34)	12 (35)	103 (29)	0.109
Hepatobiliary diseases	34 (21)	19 (32)	35 (35)	6 (18)	94 (27)	0.048
Genitourinary diseases	68 (43)	25 (42)	50 (50)	12 (35)	155 (44)	0.485
Diabetes mellitus	39 (25)	18 (31)	36 (36)	9 (27)	102 (29)	0.290
Hematological diseases	13 (8)	5 (9)	12 (12)	4 (12)	34 (10)	0.743
Solid tumors	51 (32)	26 (44)	33 (33)	13 (38)	123 (35)	0.372
Autoimmune diseases	11 (7)	3 (5)	7 (7)	2 (6)	23 (7)	0.962
Charlson CMI (mean±SD)	4.1±3.1	5.4±3.6	5.2±3.2	4.8±3.0	4.7±3.2	0.004
Recent operation (%)	31 (20)	20 (34)	24 (24)	9 (27)	84 (24)	0.116
Intra-vascular devices (%)	95 (60)	40 (68)	71 (70)	20 (59)	226 (64)	0.284
Interval from admission to onset (days, mean±SD)	17.5±25.5	29.8±48.4	27.5±35.5	25.4±44.0	23.2±35.3	0.306
Persistent bacteremia (%)	21 (13.2)	20 (33.9)	26 (25.7)	6 (17.6)	73 (20.7)	0.004
Types of bactermia by focus (%)						0.108
SABNF	78 (49)	21 (36)	34 (34)	17 (50)	150 (43)	
IVC- SAB	66 (42)	30 (51)	48 (48)	12 (35)	156 (44)	
SABWF	15 (9)	8 (14)	19 (19)	5 (14)	47 (13)	
Septic shock (%)	21 (13)	6 (10)	19 (19)	4 (12)	50 (14)	0.415
Endocarditis (%)	2 (4), (53)^b^	2 (7%), (29)^b^	5 (11), (47)^b^	0, (9)^b^	9 (7%), (138)^b^	0.113
Effective antibiotics within 48 hours after onset (%)	137 (86)	44 (75)	75 (74), (100)^b^	22 (65)	278 (79)	0.013
WBC (K/μL, mean±SD)	10.2±6.2	13.9±10.5	11.4±7.7	12.5±13.7	11.4±8.4	0.033
Hb (g/dL, mean±SD)	10.4±2.8	10.1±1.9	10.0±1.8	9.8±1.9	10.2±2.3	0.887
Platelet (K/μL, mean±SD)	157.1±102.3	180.3±108.8	177.1±111.7	172.4±147.2	168.2±110.6	0.556
CRP (mg/dL, mean±SD)	7.2±8.8, (109)^b^	9.1±8.5, (36)^b^	9.7±7.4, (69)^b^	9.8±8.8, (25)^b^	8.5±8.4, (239)^b^	0.334
Hypoalbuminemia (%)	8 (17), (46)^b^	3 (27), (11)^b^	7 (23), (31)^b^	1 (9), (11)^b^	19 (19), (99)^b^	0.679
Abnormal liver function (%)	2 (3), (72)^b^	0, (31)^b^	2 (3), (58)^b^	1 (8), (13)^b^	5 (3), (174)^b^	0.558
Impaired renal function (%)	61 (45), (137)^b^	17 (31), (55)^b^	38 (43), (89)^b^	7 (23), (31)^b^	123 (39), (312)^b^	0.065
In-hospital mortality (%)	37 (23.3)	18 (30.5)	48 (47.5)	12 (35.3)	115 (32.6)	< 0.001

Abbreviations: MSSA, methicillin-susceptible *S*. *aureus*; CA-MRSA-S, strains of community-associated methicillin-resistant *S*. *aureus*; HA-MRSA-S, strains of healthcare-associated methicillin-resistant *S*. *aureus*; Charlson CMI, Charlson co-morbidity index; SABNF, *S*. *aureus* bacteremia without identifiable focus; IVC-SAB, intra-vascular catheter-associated *S*. *aureus* bacteremia; SABWF, *S*. *aureus* bacteremia with identifiable focus other than intravenous catheter; WBC, white blood cell; Hb, hemoglobin; CRP, C-reactive protein.

^a^ *P* values here indicate for probabilities of general distributions. Post-hoc analyses show significant differences between patients with CA-MRSA-S and HA-MRSA-S bacteremia in age (*p* = 0.034), patients with MSSA and CA-MRSA-S bacteremia in respiratory disease (*p* = 0.048), patients with MSSA and HA-MRSA-S bacteremia in respiratory disease (*p* = 0.034), patients with MSSA and CA-MRSA-S bacteremia in persistent bacteremia (*p* = 0.003), patients with MSSA and CA-MRSA-S bacteremia in WBC count (*p* = 0.043), patients with MSSA and unclassified MRSA bacteremia in effective antibiotics within 48 hours (*p* = 0.017), and patients with MSSA and HA-MRSA-S bacteremia in in-hospital mortality (*p* = 0.039).

^b^The numbers in these parentheses indicate actual numbers of patients tested for the parameters, because missing data are present.

Comparisons of the manifestations among the patients with MSSA, CA-MRSA-S, HA-MRSA-S, and unclassified MRSA bacteremia are presented in Table 1. Post-hoc analysis demonstrates that the distribution of age, presence or not of underlying respiratory diseases, persistent bacteremia, receiving effective antibiotic treatment within 48 hours after the onset of SAB, WBC counts, and in-hospital mortality rates differed significantly between these groups.

Comparisons of the characteristics between the survived patients or deceased patients (in-hospital mortality) are shown in Table 2. Univariate analysis revealed that old age, the presence of solid tumors, a high Charlson co-morbidity index, a longer interval from admission to the onset of SAB, the presence of persistent bacteremia, septic shock, not receiving effective antibiotic treatment within 48 hours after the onset of SAB, thrombocytopenia, a high CRP level, bacteremia caused by HA-MRSA-S, and bacteremia caused by MRSA isolates carrying the type II or III SCC*mec* element to be associated with in-hospital mortality.

**Table 2:** The demographic, clinical, and relevant microbiological data of the 353 patients with nosocomial *S*. *aureus* bacteremia stratified by in-hospital mortality and univariate analysis for all-cause in-hospital mortality.

Variables	Survived (N = 238)	died (N = 115)	OR of univariate analysis for mortality	*p* value of univariate analysis for mortality
Age (years, mean±SD)	57.6±26.2	64.9±19.3	1.013	0.010
Gender (male to female)	86:152	46:69	1.178	0.482
Underlying diseases (%)				
Cardiovascular diseases	145 (61)	74 (64)	1.158	0.535
Respiratory diseases	67 (28)	43 (37)	1.524	0.080
Neurologic diseases	74 (31)	36 (31)	1.010	0.968
Gastrointestinal diseases	67 (28)	36 (31)	1.163	0.542
Hepatobiliary diseases	59 (24)	35 (30)	1.327	0.262
Genitourinary diseases	108 (45)	47 (41)	0.832	0.420
Diabetes mellitus	70 (29)	32 (28)	0.920	0.740
Hematological diseases	23 (10)	11 (10)	0.898	0.976
Solid tumors	63 (27)	58 (50)	2.708	<0.001
Autoimmune diseases	16 (7)	7 (6)	0.899	0.821
Charlson CMI (mean±SD)	4.0±3.1	6.1±3.1	1.232	<0.001
Recent operation (%)	54 (23)	30 (26)	1.203	0.483
Intra-vascular devices (%)	145 (61)	81 (70)	0.533	0.573
Interval from admission to onset (days, mean±SD)	19.0±30.1	31.9±41.9	1.010	0.003
Persistent bacteremia (%)	39 (16)	34 (30)	2.142	0.005
Types of bacteremia by focus (%)				
SABNF	109 (46)	41 (36)	Baseline	Baseline
IVC-SAB	101 (42)	55 (48)	1.448	0.136
SABWF	28 (12)	19 (17)	1.804	0.091
Septic shock (%)	16 (7%)	34 (30%)	5.824	<0.001
Endocarditis (%)	4 (4%), (98)^a^	5 (13), (40)^a^	3.357	0.083
Effective antibiotics within 48 hours after onset (%)	196 (83), (237)^a^	82 (71)	0.520	0.015
Abnormal WBC count (%)	130 (55)	74 (64)	1.499	0.083
Anemia (%)	118 (50)	64 (57)	1.282	0.288
Thrombocytopenia (%)	54 (23)	44 (38)	2.822	0.003
CRP (mg/dL, mean±SD)	6.8±6.7, (168)^a^	12.3±10.5, (71)^a^	1.080	<0.001
Hypoalbuminemia (%)	10 (15), (66)^a^	9 (27), (33)^a^	2.100	0.154
Abnormal liver function (%)	2 (2), (105)^a^	3 (4), (69)^a^	2.341	0.359
Impaired renal function (%)	79 (38), (207)^a^	44 (42), (105)^a^	1.169	0.523
Causative strains (%), Model 1				
MSSA	122 (51)	37 (32)	Baseline	Baseline
CA-MRSA-S	41 (17)	18 (16)	1.448	0.276
HA-MRSA-S	53 (22)	48 (42)	2.986	<0.001
Unclassified MRSA	22 (9)	12 (10)	1.799	0.147
Causative strains (%), Model 2				
No SCC*mec* (MSSA)	122 (51)	37 (32)	Baseline	Baseline
Type II SCC*mec*	22 (9)	18 (16)	2.698	0.007
Type III SCC*mec*	31 (13)	30 (26)	3.191	< 0.001
Type IV SCC*mec*	11 (5)	3 (2.6)	0.899	0.876
Type V SCC*mec*	30 (13)	15 (13)	1.649	0.174
Unknown SCC*mec* type	22 (9)	12 (10)	1.799	0.147

Abbreviations: OR, odds ratio; MSSA, methicillin-susceptible *S*. *aureus*; CA-MRSA-S, strains of community-associated methicillin-resistant *S*. *aureus*; HA-MRSA-S, strains of healthcare-associated methicillin-resistant *S*. *aureus*; Charlson CMI, Charlson co-morbidity index; SABNF, *S*. *aureus* bacteremia without identifiable focus; IVC-SAB, intra-vascular catheter-associated *S*. *aureus* bacteremia; SABWF, *S*. *aureus* bacteremia with identifiable focus other than intravenous catheter; WBC, white blood cell; Hb, hemoglobin; CRP, C-reactive protein.

^a^ The numbers in these parentheses indicate actual numbers of patients tested for the parameters, because missing data are present.

The results of the multivariate analyses are presented in Table 3. In Model 1, bacteremia caused by HA-MRSA-S [odds ratio (OR) = 2.249, *p* = 0.013], a high Charlson co-morbidity index (OR = 1.239, *p*<0.001), septic shock (OR = 7.379, *p*<0.001), thrombocytopenia (OR = 1.809, *p* = 0.047), and persistent bacteremia (OR = 2.283, *p* = 0.010) were independent risk factors associated with in-hospital mortality. In Model 2, bacteremia caused by MRSA isolates carrying the type II (OR = 2.360, *p* = 0.040) or III (OR = 2.443, *p* = 0.011) SCC*mec* element, a high Charlson co-morbidity index (OR = 1.242, *p*<0.001), septic shock (OR = 6.347, *p*<0.001), and persistent bacteremia (OR = 2.062, *p* = 0.020) were independent risk factors associated with in-hospital mortality. In Model 3, bacteremia caused by HA-MRSA-S (OR = 2.653, *p*<0.001), and a high Charlson co-morbidity index (OR = 1.228, *p*<0.001) were independent risk factors associated with in-hospital mortality. In all the three models, age (Model 1: OR = 1.009, *p* = 0.209; Model 2: OR = 1.007, *p* = 0.288; Model 3, OR = 1.005, *p* = 0.436) and effective antibiotic treatment within 48 hours (Model 1: OR = 0.564, *p* = 0.085; Model 2: OR = 0.520, *p* = 0.051; Model 3: OR = 0.646, *p* = 0.132) were not significant factors so were not retained in the regression analysis. Even adjusted with age and effective antibiotic treatment within 48 hours (keeping these two variables coercively in the regression equation of the multivariate analyses), the variables listed above remain independent risk factors associated with the in-hospital mortality.

**Table 3:** Multivariate analysis for risk factors associated with all-cause in-hospital mortality.

Variables in final model	Odds ratio	95% confidence interval		*p* value
		Lower	Upper	
Model 1				
Causative strains (using MSSA as baseline)				
CA-MRSA-S	0.998	0.453	2.200	0.996
HA-MRSA-S	2.249	1.188	4.259	0.013
Unclassified MRSA	1.223	0.466	3.210	0.683
Charlson co-morbidity index	1.239	1.139	1.348	<0.001
Septic shock	7.379	3.464	15.721	<0.001
Thrombocytopenia	1.809	1.007	3.248	0.047
Persistent bacteremia	2.283	1.214	4.292	0.010
Model 2				
Causative strains by SCC*mec* (using no SCC*mec* as baseline)				
Type II SCC*mec*	2.360	1.039	5.360	0.040
Type III SCC*mec*	2.443	1.226	4.868	0.011
Type IV SCC*mec*	0.762	0.169	3.433	0.724
Type V SCC*mec*	1.129	0.498	2.560	0.772
Unknown SCC*mec* type	1.744	0.723	4.203	0.216
Charlson co-morbidity index	1.242	1.145	1.347	<0.001
Septic shock	6.347	3.143	12.815	<0.001
Persistent bacteremia	2.062	1.123	3.788	0.020
Model 3				
Causative strains (using MSSA as baseline)				
CA-MRSA-S	1.106	0.545	2.242	0.780
HA-MRSA-S	2.653	1.515	4.646	<0.001
Unclassified MRSA	1.633	0.715	3.731	0.244
Charlson co-morbidity index	1.228	1.137	1.327	<0.001

Abbreviations: MSSA, methicillin-susceptible *S*. *aureus*; CA-MRSA-S, strains of community-associated methicillin-resistant *S*. *aureus*; HA-MRSA-S, strains of healthcare-associated methicillin-resistant *S*. *aureus*.

When all of the bacteremia caused by MRSA were pooled, the patients with MRSA bacteremia had a significantly higher in-hospital mortality rate than the patients with MSSA bacteremia in the multivariate analysis (OR = 1.707, *p* = 0.026). In this analysis, other independent risk factors for in-hospital mortality included high Charlson co-morbidity index (OR = 1.237, *p*<0.001), septic shock (OR = 6.665, *p*<0.001), and persistent bacteremia (OR = 1.936, *p* = 0.029).

When the 160 patients with bacteremia caused by either CA-MRSA-S or HA-MRSA-S were considered, the independent factors associated with in-hospital mortality are bactermia caused by HA-MRSA-S as opposed to CA-MRSA-S (OR = 2.451, *p* = 0.022), a high Charlson co-morbidity index (OR = 1.131, *p* = 0.022), septic shock (OR = 3.061, *p* = 0.026), effective antibiotics within 48 hours (OR = 0.263, *p* = 0.001), and persistent bacteremia (OR = 2.206, *p* = 0.050).

The main results of the microbiological studies are presented in Table 4. The CA-MRSA-S were more frequently susceptible than the HA-MRSA-S to ciprofloxacin, gentamicin, rifampin, and SXT. The susceptibilities to erythromycin and clindamycin were both low in CA-MRSA-S (15.7% and 33.9%, respectively) and HA-MRSA-S (23.8% and 26.7%, respectively). All of the isolates were susceptible to vancomycin. Four MRSA isolates were not susceptible to daptomycin (two each of CA-MRSA-S and HA-MRSA-S). Another one isolate of CA-MRSA-S was not susceptible to linezolid. None of the MRSA isolates carried the type I SCC*mec* element. Sixty-one isolates of HA-MRSA-S carried the type III SCC*mec* element, and 39 isolates of CA-MRSA-S carried the type V SCC*mec* element. Fifty-eight MRSA isolates had a vancomycin MIC of 2mg/L, and of these, four isolates belonged to CA-MRSA-S and the other 54 isolates belonged to HA-MRSA-S. Therefore, a high-vancomycin MIC had a high level of collinearity with HA-MRSA-S in the present study; hence, entering both variables into the final multivariate analysis model simultaneously was unsatisfactory, because it led to divergent results when estimating the regression coefficients in statistical analyses. However, if vancomycin MIC levels was considered as a variable, replacing the variable of molecular classification of MRSA (CA-MRSA-S, HA-MRSA-S, unclassified MRSA), in the multivariate analysis on risk factors associated all-cause in-hospital mortality among MRSA bacteremic patients, it remains insignificant (OR = 1.751, *p* = 0.115).

**Table 4:** Drug susceptibilities (%) and carriage of SCC*mec* elements (%) of the 261 *S*. *aureus* blood isolates.

	Susceptible rates to various antibiotics (%)										SCC*mec* element (%)				
	Cip	Clin	Dap	Ery	Gen	Lin	Rif	Tet	SXT	Van	I	II	III	IV	V
MSSA (N = 101)	88	91	100	76	85	99	100	63	94	100	0	0	0	0	0
CA-MRSA-S (N = 59)	75	34	97	16	63	98	100	49	95	100	0	0	0	24	66
HA-MRSA-S (N = 101)	26	27	98	24	29	100	80	26	42	100	0	40	60	0	0

Abbreviations: Cip, ciprofloxacin; Clin, clindamycin; Dap, daptomycin; Ery, erythromycin; Gen, gentamicin; Lin, linezolid; Rif, rifampin; Tet, tetracycline; SXT, trimethoprim/sulfamethoxazole; Van, vancomycin; MSSA, methicillin-susceptible *S*. *aureus*; CA-MRSA-S, strains of community-associated methicillin-resistant *S*. *aureus*; HA-MRSA-S, strains of healthcare-associated methicillin-resistant *S*. *aureus*.

## Discussion

In our present study, the all-cause in-hospital mortality rates in patients with nosocomial *S*. *aureus*, MSSA, and MRSA bacteremia were similar to prior studies [26]. The patients with MRSA bacteremia had a significantly higher in-hospital mortality than those with MSSA bacteremia. However, when the MRSA bacteremia was stratified into HA-MRSA-S, CA-MRSA-S, and unclassified bacteremia, only the patients with HA-MRSA-S, but not CA-MRSA-S, bacteremia had a significantly higher in-hospital mortality rate compared with the patients with MSSA bacteremia. Other risk factors associated with in-hospital mortality were a high Charlson co-morbidity index (Model 1, 2, and 3), septic shock (Model 1 and 2), thrombocytopenia (Model 1), and persistent bacteremia (Model 1 and 2). These associations remain present even after adjustment with patients’ age and whether receiving effective antibiotic treatment within 48 hours after the onset of bacteremia.

The effect of methicillin resistance on the outcome of patients with SAB has been reported in many studies [26]. Although the findings from most studies show that methicillin resistance is independently associated with the mortality of SAB, several other studies have showed different results [26]. The reasons for the different results may be related to the differences in the clinical settings (bacteremia in community setting, healthcare-associated settings, or both), the patients’ characteristics, the end-points evaluated, and the enrollment of patients with concomitant infections, which would lead to great difficulties in determining the attributable factors for mortality [13, 26–30]. The present study enrolled patients with nosocomial SAB and without concomitant infections; hence, the patient population was more homogenous and the outcomes of the SAB were less confounded by other infections. In addition, the prevalence of MRSA in the hospital settings is high in Taiwan [17]; therefore, patients with HAIs who are at risk of gram-positive bacterial infections are usually administered glycopeptides empirically. In the present study, a much higher proportion of patients with MRSA bacteremia received effective antibiotic treatment within 48 hours after the onset of SAB compared with that of an earlier report, which reported that only 45% of patients with MRSA bacteremia received effective empirical treatment [31]. This would reduce the impact of “delay in effective antibiotic treatment” on the outcome of patients with MRSA bacteremia in the comparison with patients with MSSA bacteremia, which in turn would make our analyses less confounded by treatment effect.

Previous reports emphasize that there were differences in the distributions of hosts’ characteristics between those with nosocomial/healthcare-associated CA-MRSA-S and HA-MRSA-S bacteremia, and CA-MRSA-S bacteremia was associated with a better outcome compared to HA-MRSA-S bacteremia [11,12, 25]. Our study’s findings echoed those from previous reports because our findings demonstrated that patients with CA-MRSA-S bacteremia were younger, had lower rates of underlying cardiovascular disease, and they had better outcomes than the patients with HA-MRSA-S bacteremia. Chen et al reported that traditional hospital strains, not community strains, MRSA were associated with an adverse outcome compared with MSSA among the patients with community-onset SAB [15]. Our results support their findings and provide an evidence that this phenomenon is also noted among patients with nosocomial SAB.

For Model 2 multivariate analysis, bacteremia caused by MRSA carrying the type II or type III SCC*mec* element, but not the type IV or V, is independently associated with in-hospital mortality compared with that caused by MSSA. This result is similar to that of Model 1 analysis. Ganga et al had reported a similar finding that SAB caused by MRSA carrying the type II, but not type IVa, SCC*mec* element, was associated with all-cause in-hospital mortality compared with that caused by MSSA [16]. In that study, no MRSA isolates were carrying the type III or V SCC*mec* element. Our results provided additional information that SAB caused by MRSA isolates carrying the type II or III SCC*mec* element was also associated with in-hospital mortality, but that by MRSA isolates carrying the type IV or V SCC*mec* element did not.

One reason why MRSA isolates carrying type II or III SCC*mec* element (HA-MRSA-S) were associated with in-hospital mortality might be that these isolates carried virulent factors. Previous study reported that MRSA isolates with type II or III SCC*mec* element could carry various virulent genes, including *hla*, *hlb*, *seg*, *sei*, *tst*, *sec*, *sej*, *fnbA*, *clfA*, *clfB*, *ebpS*, *bbp*, and *cna*, which in turn might result in more severe diseases in humans [32]. Virulent factors were not tested in our present study. Further investigation is needed to validate this possible association.

Other possible confounding factor was the vancomycin MIC levels of the MRSA isolates. However, the vancomycin MIC level was not identified as a risk factors for in-hospital mortality in the present study. Several prior reports showed that high- but susceptible- vancomycin MICs, specifically, ≥1.5 mg/L that were determined using Etest, or ≥2 mg/L that were determined using broth dilution method, increased the mortality of patients with vancomycin-treated MRSA bacteremia [33–35]. However, several other reports did not find this association [36–38]. A recent major meta-analysis concluded that for patients with bacteremia caused by MRSA susceptible to vancomycin and treated by vancomycin, there was no statistically significant difference in mortality rates of patients whose MRSA isolates had higher vancomycin MICs compared with those of patients whose MRSA isolates had lower vancomycin MICs. However, all of the studies enrolled for analysis were observational ones [39]. More evidence is needed to make any conclusion regarding the impact of vancomycin MIC.

Other risk factors associated with in-hospital mortality that were identified in Model 1 and/or Model 2 of the current study included a high Charlson co-morbidity index, septic shock, thrombocytopenia, and persistent bacteremia. All of these factors are either surrogate indicators of the host’s condition or indicators of the burden of disease and its severity, and their associations with in-hospital mortality concur with previous findings [26].

Although many previous studies, and also the Model 1 and 2 analyses in our present study, considered variables of SAB severity, such as septic shock, thrombocytopenia, and persistent bacteremia, as significant factors associated with SAB outcomes, these variables were downstream events following the onset of SAB. They might introduce additional bias when they were retained in the multivariate analyses that were constructed to infer whether patients with SAB caused by different MRSA strains were associated with different outcomes in comparison to those by MSSA [40]. In Model 3, these variables were left out to avoid the potential bias. The results still showed that only the patients with HA-MRSA-S, but not CA-MRSA-S, bacteremia had a significantly higher in-hospital mortality rate compared with the patients with MSSA bacteremia.

The results of the drug susceptibility tests of the present study were similar to those from previous Taiwanese studies [8]; however, resistance to daptomycin and linezolid was noted. Daptomycin resistance was noted in isolates of CA-MRSA-S and HA-MRSA-S, and linezolid resistance was noted in isolates of CA-MRSA-S only. Although the resistance rates were low, continuous monitoring of the trends relating to the susceptibilities of MRSA isolates to daptomycin and linezolid in MRSA isolates is necessary, because daptomycin and linezolid are important alternatives to glycopeptides for the treatment of MRSA infections [41].

The serum vancomycin levels were not available in our study, because this was a retrospective study, and some patients were administered teicoplanin rather than vancomycin. However, inadequate (low) serum vancomycin levels would have adversely affected the outcomes associated with vancomycin-treated MRSA bacteremia. Therefore, even if the serum vancomycin levels had been available, they would not have affected the findings of similar outcomes for CA-MRSA-S and MSSA bacteremia. Whether the serum vancomyin level is a confounding factor for the inferior outcomes of HA-MRSA-S bacteremia needs further investigation.

There are two limitations of our present study. First, this was a retrospective study; therefore, it was inevitable that there would be missing data and potential information bias. Second, caution is advised when extrapolating our findings to other institutions, because this study only involved two hospitals.

In conclusion, the findings from this study show that compared with MSSA bacteremia, HA-MRSA-S, but not CA-MRSA-S, bacteremia is an independent risk factor for all-cause in-hospital mortality in patients with nosocomial SAB. Our results emphasize that it might be necessary to stratify MRSA isolates into HA-MRSA-S and CA-MRSA-S while performing outcome analyses of patients with MRSA bacteremia. In addition, resistance to daptomycin and linezolid was noted among the MRSA isolates in Taiwan. Hence, further surveillance of the drug susceptibilities of clinical MRSA isolates is necessary.
